# Role of Eosinophils in Intestinal Inflammation and Fibrosis in Inflammatory Bowel Disease: An Overlooked Villain?

**DOI:** 10.3389/fimmu.2021.754413

**Published:** 2021-10-19

**Authors:** Inge Jacobs, Matthias Ceulemans, Lucas Wauters, Christine Breynaert, Séverine Vermeire, Bram Verstockt, Tim Vanuytsel

**Affiliations:** ^1^ Department of Microbiology, Immunology and Transplantation, Allergy and Clinical Immunology Research Group, Katholieke Universiteit Leuven, Leuven, Belgium; ^2^ Department of Chronic Diseases and Metabolism, Translational Research Center for Gastrointestinal Disorders (TARGID), Katholieke Universiteit Leuven, Leuven, Belgium; ^3^ Department of Gastroenterology and Hepatology, University Hospitals Leuven, Leuven, Belgium; ^4^ Department of General Internal Medicine, Allergy and Clinical Immunology, University Hospitals Leuven, Leuven, Belgium

**Keywords:** eosinophils, IBD, inflammation, fibrosis, gastrointestinal disorders

## Abstract

Eosinophils are leukocytes which reside in the gastrointestinal tract under homeostatic conditions, except for the esophagus which is normally devoid of eosinophils. Research on eosinophils has primarily focused on anti-helminth responses and type 2 immune disorders. In contrast, the search for a role of eosinophils in chronic intestinal inflammation and fibrosis has been limited. With a shift in research focus from adaptive to innate immunity and the fact that the eosinophilic granules are filled with inflammatory mediators, eosinophils are becoming a point of interest in inflammatory bowel diseases. In the current review we summarize eosinophil characteristics and recruitment as well as the current knowledge on presence, inflammatory and pro-fibrotic functions of eosinophils in inflammatory bowel disease and other chronic inflammatory conditions, and we identify research gaps which should be covered in the future.

## 1 Introduction

Inflammatory bowel diseases (IBD), further subdivided into Crohn’s disease (CD) and ulcerative colitis (UC), are idiopathic, heterogeneous disorders characterized by a relapsing and remitting disease course (1). Both disorders are believed to result from an inappropriate immune response towards the intestinal microbiota in genetically predisposed patients (1). Although certain genetic risk factors, e.g. polymorphisms in *nucleotide-binding oligomerization domain-containing 2* (*NOD-2*) and *autophagy related protein like 1* (*ATGL1)* have been identified, the exact pathogenesis remains elusive (1, 2).

An excessive immune reaction occurs in response to a loss of the epithelial barrier integrity and damage to tissues, thereby further leading to inflammation (3, 4). This repetitive inflammatory response in IBD patients is considered to contribute to the development of excessive extracellular matrix (ECM) deposition resulting in intestinal fibrosis, especially in CD, due to its transmural character. Stricture formation through fibrosis resulting in complications like intestinal obstruction is the most common indication for surgery in CD patients (5).

Intestinal fibrosis manifests itself only in previously or actively inflamed regions of the gastrointestinal (GI) tract, indicating that inflammation is a *sine qua non* condition to develop fibrosis (6, 7). Hence, research primarily focused on the inflammatory process, but not on the resulting fibrosis. Repetitive inflammatory injury to the intestine can result in the release of growth factors, thereby stimulating fibroblast proliferation and the differentiation from fibroblasts to myofibroblasts which will ultimately result in excessive deposition of ECM (8). However, the characterization of the key immune cells and their mediators involved in gut fibrogenesis awaits further investigation.

Most studies on the pathogenesis of inflammatory disorders have focused on excessive adaptive immune responses, although recently the focus has shifted to innate immunity (9). In this context, the search for a potential role for involvement of eosinophils in inflammation and fibrosis recently became relevant again (10, 11). Already several decades ago, eosinophils were identified as important contributing cells to the immune cell infiltration in IBD, e.g. with the inclusion of eosinophil infiltration in the lamina propria in the Geboes histological score for UC (12, 13). Furthermore, eosinophilia-associated basal plasmacytosis is considered a hallmark in early diagnosis of IBD and strongly correlated with histological diagnosis (14). Additionally, important eosinophil infiltration in the lamina propria of colonic biopsies in UC patients was previously demonstrated to be the most significant predictor of poor response to medical therapy (15). Moreover, the extensive presence of pre-formed mediators in the eosinophilic granules, known to be involved in inflammation or fibrosis, makes these innate immune cells particularly interesting in the context of fibrostenosis in IBD and in the search of novel treatment targets (9). Although several reports already suggested the eosinophilic granulocytes to be associated with increased levels of inflammation and fibrosis development, a causal role or mechanism has not yet emerged.

In the current review we summarize eosinophil characteristics and recruitment as well as the current knowledge on presence, inflammatory and pro-fibrotic function of eosinophils in IBD and other chronic inflammatory conditions, and we identify research gaps which should be covered in the future.

## 2 Gastrointestinal Eosinophils

### 2.1 General Characteristics

**Figure 1:**
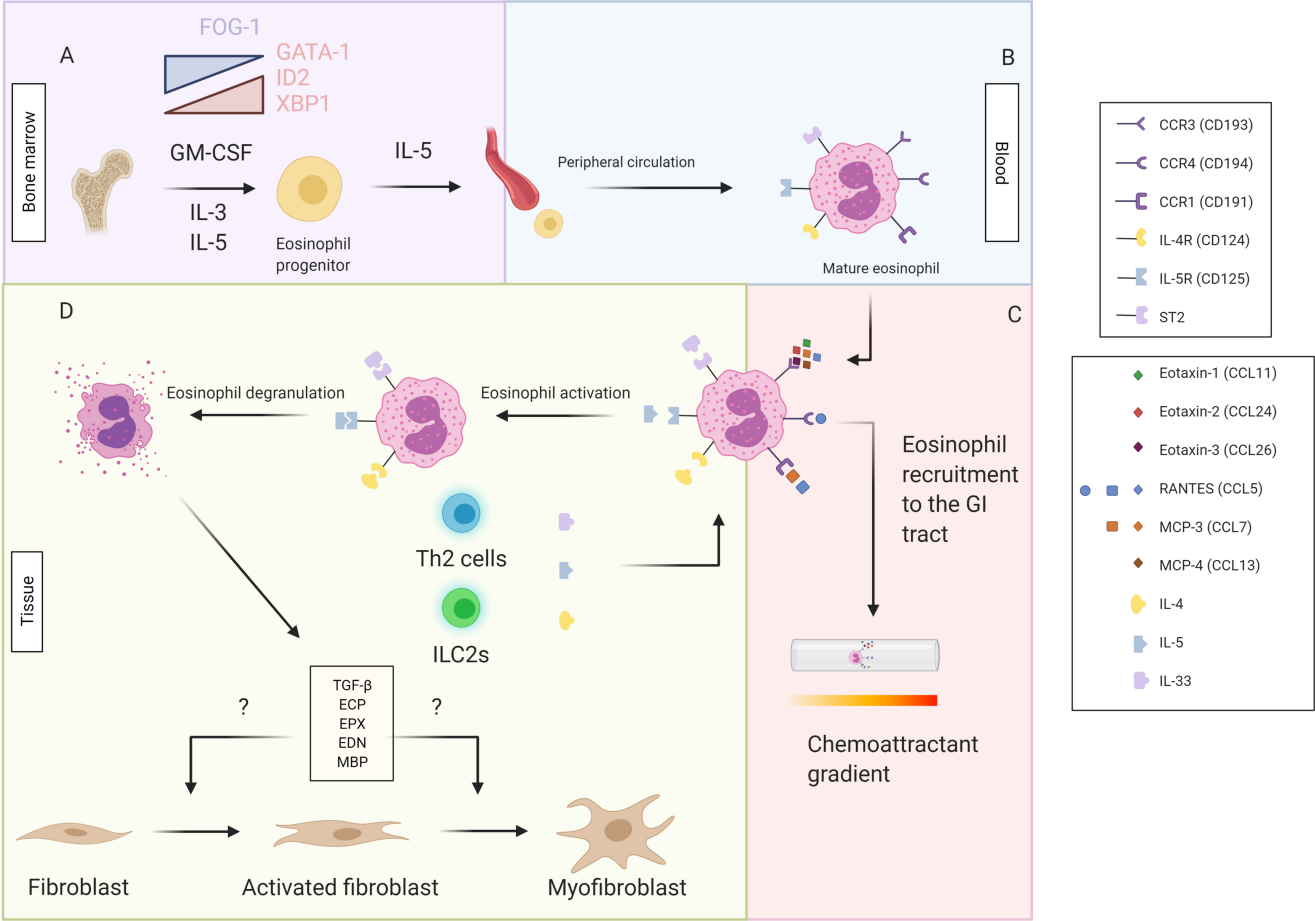
Pluripotent hematopoeitic stem cells differentiate from the bone marrow to eosinophil progenitors in response to GM-CSF, IL-3, IL-5, a decrease in transcription factor FOG-1 and increased presence of the transcription factors GATA-1, ID2 and XBP1 **(A)**. Under the influence of IL-5 the eosinophil progenitor will be released in the peripheral circulation and further develop into mature eosinophils in the blood **(B)**. By the binding of the chemoattractants (eotaxin-1, eotaxin-2, eotaxin-3, MCP-3, MCP-4 and RANTES) to the chemoattractant receptors (CCR1, CCR3 and CCR4) a chemoattractant gradient is created and the mature eosinophils are recruited to the GI tract **(C)**. The binding of the cytokines IL-4, IL-5 and IL-33, primarily produced by the Th2 cells and ILC2s, to their respective receptor (IL-4R or CD124, IL-5R or CD125 and ST2) causes eosinophil activation and subsequent degranulation releasing TGF-ß, ECP, EPX, EDN and MBP. These factors possibly influence fibroblast activation and differentiation from fibroblasts to myofibroblasts **(D)**. This figure was created via biorender.com.

Eosinophils are leukocytes that reside in the lamina propria of the gastrointestinal (GI) tract (16, 17). They are normal resident immune cells in the entire GI tract, with exception of the esophagus, where eosinophils are only present under inflammatory conditions such as gastro-esophageal reflux disease and eosinophilic esophagitis (18). Under the influence of interleukin (IL)-3, IL-5 and granulocyte-macrophage colony stimulating factor (GM-CSF), accompanied with a decrease in transcription factor FOG-1 and increased presence of the transcription factors GATA-1, ID2 and XBP1, eosinophils differentiate from pluripotent hematopoietic stem cells in the bone marrow to mature eosinophils (**Figure 1A**) (19–22). In response to IL-5, eosinophils are released into the peripheral circulation (**Figure 1B**), after which they can migrate to the GI tract after binding of chemoattractant molecules, in particular C-C motif ligand 11 (CCL11, eotaxin-1), CCL24 (eotaxin-2), CCL26 (eotaxin-3), CCL5 [Regulated upon activation, normal T cell expressed and secreted (RANTES)], CCL7 [Monocyte chemoattractant protein-3 (MCP-3)] and CCL13 (MCP-4), to their C-C chemokine receptors (CCR) (CCR1, CCR3 and CCR4) (**Figure 1C**) (23). The activation of these receptors triggers both eosinophil recruitment and activation, and thereby induces the production of several cytokines [IL-4, IL-5, IL-13, interferon-gamma (IFN-γ) etc.] and chemokines (CCL3, CCL5, CCL11 etc.) (20). Upon stimulation with cytokines, e.g. IL-4, IL-5 and IL-13, eosinophils will become activated (**Figure 1D**) (24). Eosinophil activation causes degranulation, possibly resulting in damage to the tissue by the release of, amongst others, toxic oxygen radicals, eosinophil cationic protein (ECP) and transforming growth factor β (TGF-β) (11, 25). Eosinophil degranulation therefore contributes to the inflammatory process, in synergy with other inflammatory cells. Among those, the most important ones are Th2 lymphocytes which express the CCR3 membrane receptor and cluster with eosinophils during inflammation (26). It was long believed that eosinophils worked purely as effector cells of the Th2 immune reaction. More recently however, it was discovered eosinophils have their own functionality while still being strongly intertwined with the Th2 lymphocytes (27). In this context, Th2 lymphocytes produce IL-4, IL-5, IL-13 and eotaxins and thereby contribute to the activation and recruitment of eosinophils (28). Eosinophils, on the other hand, produce IL-4 and IL-5, thereby stimulating differentiation of naïve Th0 to Th2 lymphocytes and stimulating Th2 lymphocytes as well (28).

### 2.2 Eosinophil Recruitment

Eosinophil recruitment to the GI tract occurs during active inflammation in IBD. In this process, chemoattractant molecules bind to their receptors present on the eosinophil membrane. Besides playing a pivotal role in eosinophil recruitment, the chemoattractant molecules partly serve as eosinophil activators as well (11). Below, we will provide an overview of the known eosinophil chemotaxis pathways.

#### 2.2.1 Eotaxin-1, -2 and -3 – CCR3 Axis

Eotaxin is a potent eosinophil chemoattractant, secreted by eosinophils, macrophages, epithelial cells, mast cells, basophils, Th2 lymphocytes and fibroblasts (29). Eotaxin-1 is believed to be a pivotal chemotactic factor and is constitutively expressed in the small intestine and colon (30–34). Besides its binding capacity to CCR3, eotaxin-1 can furthermore bind to the receptors CCR2, CCR5 and with high affinity to CXCR3, with the latter possibly acting as a decoy receptor by sequestering eotaxin-1 (35, 36). Eotaxin-1 was first established in a guinea-pig model of allergic airway inflammation (37–39). Eotaxin-2 and -3 were later discovered to carry similar functionality concerning eosinophil recruitment (40, 41).

Colonic *eotaxin-1,-2 and -3* and *CCR3* mRNA expression levels in IBD patients are significantly increased compared to healthy controls (42). Furthermore, serum and tissue eotaxin-1 protein levels correlated with IBD disease severity and eosinophil infiltration (16, 17). The crucial role of eotaxin-1 was confirmed in eotaxin deficient mice, which showed impaired eosinophil recruitment to the colon (42, 43).

Studies in eotaxin-1 deficient mice, however, revealed the chemoattractant not to be essential for the development of airway eosinophilia (44). Moreover, *eotaxin-1* knockout mice had only partial eosinophil depletion indicating other chemokines might overcome this deficiency and eotaxin-1 alone is not sufficient to support eosinophil recruitment (45). It is therefore believed that eotaxin-1 mediated eosinophil recruitment is maintained by Th2 lymphocytes by generating IL-4 and IL-5, thereby serving as eosinophil growth and stimulating factors (46).

#### 2.2.2 RANTES, MCP-3 and MCP-4 Mediated Chemotaxis

Besides binding eotaxin-1, 2 and 3, the chemoattractant receptor CCR3 similarly binds RANTES, MCP-3 and MCP-4, resulting in eosinophil chemotaxis (47–49).

Apart from being an eosinophilic chemoattractant, RANTES is a chemotactic for T lymphocytes and basophils as well. The chemokine plays an active role in leukocyte recruitment to inflammatory sites and, together with IL-2 and IFN-gamma, released by T-lymphocytes, it is also responsible for proliferation and activation of natural killer (NK) cells (47). Other than binding to the CCR3 receptor, RANTES also has a binding affinity to CCR1 and CCR4 (48). Protein and mRNA levels of RANTES were demonstrated to be elevated in both UC and CD patients (49).

MCP-3 does not only attract eosinophils, but also functions as a monocyte and neutrophil chemoattractant and regulates macrophage functioning. Apart from binding to CCR3, MCP-3 can also bind to CCR1 (48). Increased *MCP-3* expression has been associated with inflammatory diseases, such as allergic inflammation (50). *In vivo*, MCP-3 antibody mediated neutralization in mouse lung derived endothelial cells resulted in significantly decreased eosinophil accumulation, indicating that MCP-3 is an important and potent chemotactic factor (51).

MCP-4 is a chemoattractant for several cells such as eosinophils, basophils, monocytes, macrophages, immature dendritic cells and T-lymphocytes (48). This chemokine is considered to play a pivotal role in many chronic inflammatory diseases, including allergic airway inflammation and rheumatoid arthritis, by recruiting several cell types to the inflamed tissue followed by activation (52).

Because of the variety of chemoattractants, all with their unique and common features, it is likely that eosinophil chemotaxis is not induced by binding of a single chemoattractant to its receptor, but rather a complex interplay of multiple of the factors described (48).

## 3 Role of Eosinophils in Inflammation

### 3.1 Eosinophil Activation

**Figure 2:**
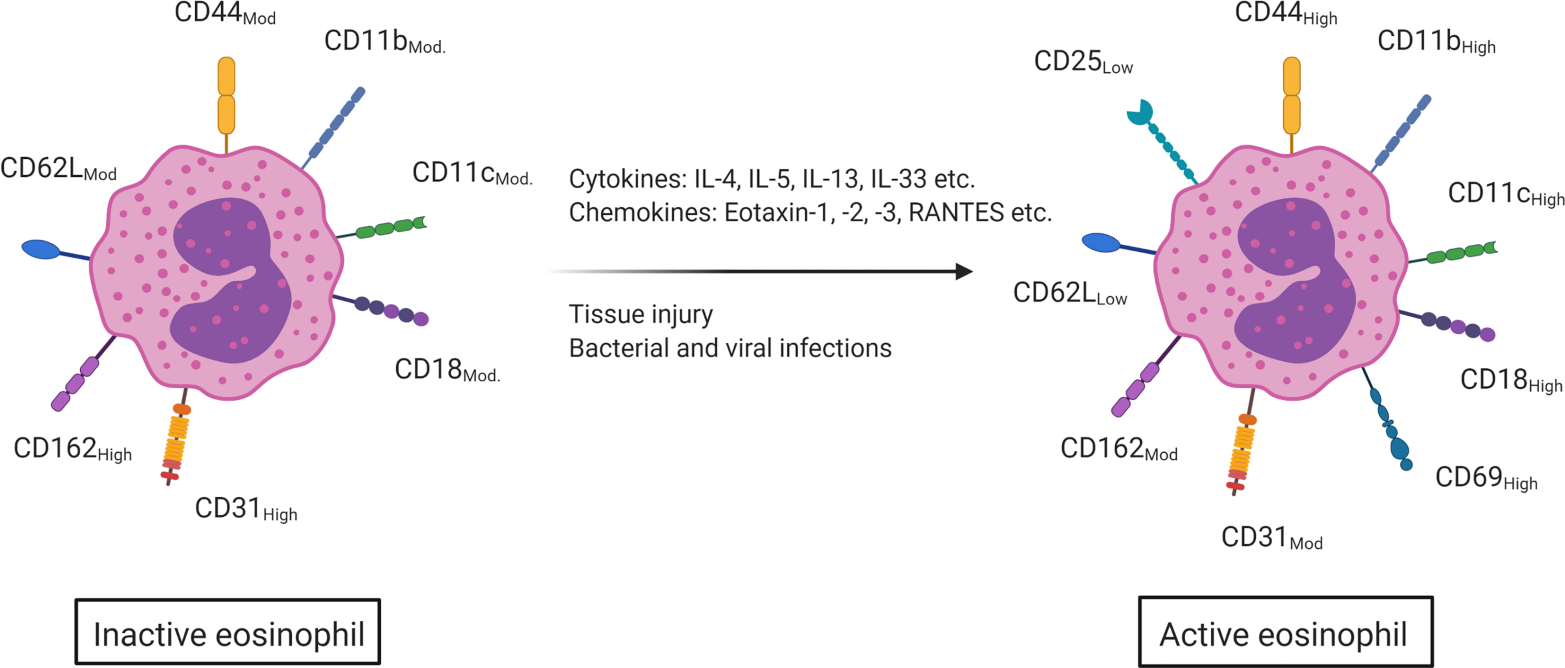
Upon contact with several cytokines (IL-4, IL-5, IL-13, IL-33, etc.), chemokines (eotaxin-1,2 and -3, RANTES etc.) and *via* tissue damage and bacterial and viral infections, eosinophils will become activated. This activation is marked by an increased surface expression of CD18, CD44, CD11b and CD11c (moderate to high expression). CD25 and CD69 are not present on inactive eosinophils, but are on active eosinophils (low and high expression respectively). CD162 and CD31 on the other hand are highly expressed on inactive eosinophils but only moderately on active eosinophils and CD62L is moderately expressed on inactive eosinophils but becomes lowly expressed once the eosinophil is activated. This figure was created *via* biorender.com.

Upon stimulation, activated eosinophils will degranulate and subsequently release their preformed granular content in the environment. Several eosinophil activating mechanisms have been described such as tissue damage, bacterial and viral infections, the binding of cytokines (IL-4, IL-5, IL-13, IL-33, etc.) and the binding of certain chemokines (eotaxin, RANTES, etc.) (53, 54). In humans, this eosinophil activation is characterized by an upregulated expression of the surface markers CD44, CD11c, CD11b and CD18. CD31 and CD162, however, are highly expressed on inactive eosinophils, but become moderately expressed upon activation. While CD25 and CD69 are not present on quiescent eosinophils, they are, respectively, lowly and highly expressed on activated eosinophils. Lastly, the surface marker CD62L is moderately expressed on eosinophils and becomes lowly expressed upon activation (**Figure 2**) (11, 55). As eosinophil activation is strongly dependent on the cytokine milieu, these markers can help enlighten the not fully understood role of eosinophils in intestinal inflammation (56).

#### 3.1.1 IL-4

IL-4 is a cytokine that is mainly produced by basophils, mast cells, T-lymphocytes, type 2 innate lymphoid cells (ILC2s), eosinophils and neutrophils (57). This cytokine drives differentiation from naïve Th0 to Th2 lymphocytes, which in turn will produce IL-4, thereby creating a positive feedback loop, thereby further enhancing the differentiation of Th2 lymphocytes (58). This pro-inflammatory cytokine is also known to stimulate eosinophil transmigration across the endothelium and the differentiation of Th2 lymphocytes, resulting in cytokine release (59, 60). By increasing eotaxin expression, IL-4 also promotes eosinophil accumulation and eosinophil chemotaxis (61). IL-4 has been linked to several inflammatory disorders, such as asthma and allergic inflammation. The role of IL-4 has already been investigated in the pathogenesis of IBD, where it is suggested to play a pivotal role in inflammation and immune response activation, mainly in UC patients in whom increased expression has been shown (62). Indeed, IL-4 deficiency can prevent the development of colitis in *IL-10* knock out mice, which spontaneously develop colitis (63). Additionally, the dextran sodium sulphate (DSS) induced colitis and T cell transfer model also suggest that IL-4 can promote colitis (64–66). Recently, an IL-4/IL-13 dual antagonist was developed and evaluated in a murine model of oxazolone-induced colitis, where it showed to ameliorate overall disease activity (67, 68). IL-4 and IL-13 can also be targeted trough a shared receptor, comprising the IL-4Rα and IL-13α1 chains (69). In this model, blocking IL-4 and IL-13 ameliorated disease severity (70–72). Mice lacking IL-4Rα did not develop disease in this model, further indicating a potential role for IL-4 in the development of colitis and inflammation (73). In contrast, *IL-4* mRNA expression levels in CD patients’ intestinal tissue were reduced, corresponding to lower numbers of IL-4 producing cells in mucosal biopsies (74).

#### 3.1.2 IL-5

IL-5 is a chemotactic agent which promotes the differentiation of eosinophils in the bone marrow and can activate eosinophils. In addition, eosinophils produce and secrete IL-5 upon degranulation, thereby promoting their own differentiation and activation, and contributing to their own expansion (75). IL-5 is mainly produced by Th2 lymphocytes and ILC2s, and in lower quantities by NKT cells, mast cells and eosinophils (76–80). ILC2s contribute to the activation of eosinophils by producing IL-4, IL-5, also shown to synergize with eotaxins, and IL-13 (81–83). Specific inhibition of IL-5, by mepolizumab or reslizumab, or specifically blocking the IL-5 receptor, by benralizumab, has been shown to attenuate the type 2 immune response and overall disease severity of eosinophilic asthma patients indicating an important role of IL-5 in eosinophil related disorders (84).

During active inflammation, eosinophils increase IL-4, IL-5 and IL-13 expression, indicating a shift to the Th2 response. Elevated IL-5 levels were found in rectal perfusion fluid from UC patients (85). Mice with DSS induced colitis receiving anti-IL-5 treatment exhibited lower eosinophil expression, more severe weight loss and higher hemoccult scores indicating that IL-5, and eosinophils, may possibly play a protective role in colitis development as well (86). A case report describing a beneficial role of benralizumab in an UC patient shows additional evidence for an advantageous effect of blocking the IL-5 receptor in UC patients (87).

#### 3.1.3 IL-13

The cytokine IL-13 is produced by Th2 lymphocytes, CD4 cells, NKT cells, mast cells, basophils and eosinophils (88). It has been linked to airway hyperresponsiveness and fibrosis development before, as a mediator of allergic inflammation, and therefore linked to diseases such as asthma (88).

IL-13 and IL-4 share some functionality due to a shared receptor, formed by IL-4Rα and IL-13Rα1. Activation of this shared receptor results in STAT6 signaling and stimulation of the type 2 immunity (89). IL-13 can also bind to IL-13Rα2, which acts as a decoy receptor and therefore inhibits IL-13 signaling (90, 91). IL-13 binds IL-13Rα2 with an affinity about 400-fold higher than IL-4Rα/IL-13Rα1, thereby inhibiting STAT6 signaling and dampening the subsequent type 2 immunity response (92). Other studies, however, do suggest a signaling functionality for IL-13Rα2. Strober et al. described IL-13Rα2 signaling to result in TGF-β1 production thereby possibly providing a contribution to fibrosis in a model of bleomycin induced pulmonary fibrosis and oxazolone colitis. Later, this functionality was proven in a trinitrobenzene sulfonic acid (TNBS) colitis model as well (93).

*IL-13Rα2* knockout mice were protected from the induction of colitis in a DSS induced colitis model. This was confirmed by IL-13Rα2 antibody mediated neutralization in 8-12-week-old BALB/c mice which showed significant amelioration in colon health, based on colon pathology score and colon length, compared to wild type (wt) mice after DSS induced colitis (92). However, a previous report demonstrated that *IL-13Rα2* knock out mice were not protected from colitis development, but recovered and restored the mucosal layer faster (91). Elevated *IL13Rα2* mRNA expression levels in mucosal biopsies from both UC as CD patients during active disease have been reported and have been suggested as a potential biomarker for anti-TNF non-responsiveness (94–96). Although IL-13 has been implicated in the inflammatory response in UC patients, results from clinical trials are disappointing: tralokinumab and anrukinzumab, a human and humanized monoclonal anti-IL13 antibody respectively, did not show any therapeutic benefit (62). Therefore, it is still unclear which role IL-13 plays in the development of IBD, but it seems likely that it could serve as a potential therapeutic target in IBD (70, 97).

#### 3.1.4 IL-33

IL-33 is a pro-inflammatory cytokine secreted by several intestinal cells such as ILC2s, Th2 lymphocytes, epithelial cells, etc. that binds to suppression of tumorigenicity 2 (ST2), present on the eosinophilic membrane, and thereby activating the ST2/IL-33 signaling pathway (98). Upon epithelial damage IL-33 will be released and can directly expand ILC2s, Th2 cells and eosinophils. *Via* the production of IL-5 and IL-13 and the production of IL-4 and IL-5, the ILC2 and Th2 cells, respectively, can furthermore contribute to the expansion of the eosinophil population (99).

Several studies already proposed a potential involvement for IL-33 in the development of colitis: activated eosinophils, together with increased colonic *IL-33* mRNA expression levels which correlated with increased colonic *eotaxin-1* expression have been demonstrated in UC patients (100). In both intestinal biopsies from IBD patients as in the colon of SAMP/YitFc mice, which spontaneously develop colitis resembling human CD, ST2/IL-33 signaling caused an eosinophilic infiltration and activation coinciding with a Th2 mediated immune response resulting in the release of IL-4, IL-5 and IL-13 (101–103). Antibody mediated blocking of ST2 in these SAMP/YitFc mice diminished the production of Th2 cytokines, and decreased eosinophil recruitment to the ileum (102). In addition, *ST2* knockout in C57BL/6 mice alleviated disease symptoms. This was confirmed in a C57BL/6 mouse model with antibody-mediated blocking of ST2 (104).

### 3.2 Eosinophil Degranulation

Upon eosinophil activation and subsequent eosinophil degranulation, toxic substances can be released into the environment. Release of the eosinophil specific proteins eosinophil cationic protein (ECP), eosinophil peroxidase (EPO), eosinophil derived neurotoxin (EDN) and eosinophil major basic protein (MBP) were described to cause tissue damage *via* its cytotoxic activity, resulting in the destruction of the epithelial layer and thereby contributing to, amongst others, airway damage and possible lung dysfunction (105). Furthermore, the protein TGF-β1, released from the eosinophil granules, has been described to contribute to inflammation and fibrosis as well (106).

MBP is located in the core of the eosinophilic granule while ECP, EPO and EDN are stored in the surrounding matrix (107). Eosinophil degranulation occurs *via* four different mechanisms; classical exocytosis, compound exocytosis, piecemeal degranulation and cytolysis (107). It is also known that asthmatic patients’ eosinophils tend to produce more reactive oxygen species compared to healthy controls. Nitric oxide levels, believed to be a marker for the level of eosinophilic inflammation in the lower airways, are elevated in bronchial asthmatic patients and are used in diagnosis of asthma (107).

The contribution of these proteins in the development of intestinal inflammation are discussed below.

#### 3.2.1 Transforming Growth Factor β1 (TGF-β1)

TGF-β is a cytokine produced by fibroblasts, epithelial cells and immune cells (108). Three different human isoforms exist: TGF-β1, which is the most abundant form in the GI tract, TGF-β2 and TGF-β3 (109).

The role of TGF-β1, present in the eosinophilic granules, in acute intestinal inflammation is still elusive with contradictory results in the literature (110). While some studies reported increased TGF-β1 levels during active inflammation, others reported decreased levels or no significant differences at all (111, 112). In addition, TGF-β1 deficient mice spontaneously developed colitis (113, 114). Although TGF-β has been linked to inflammation before, its more important role is attributed to chronic inflammation and subsequent fibrosis which is discussed in detail in the next section (115).

#### 3.2.2 Eosinophil Cationic Protein (ECP)

Upon eosinophil degranulation, the eosinophil specific ECP, also known as ribonuclease 3, is released. The protein with a molecular weight ranging from 18 to 22 kDa is encoded by the *Ribonuclease A family member 3* gene (116). ECP can damage cell membranes by the formation of pores into transmembrane channels through which toxic molecules can enter the cell (117, 118). Eosinophils house large amounts of ECP that are released upon degranulation and therefore no *de novo* synthesis is required at the time of degranulation (118).

Patients with active CD or UC had elevated serum ECP levels compared to healthy individuals or IBD patients in remission (119). This study furthermore revealed faecal ECP (fECP) to be elevated in both CD and UC patients. The diagnostic accuracy for differentiating IBD patients with active from inactive disease however was lower for fECP compared to fecal calprotectin (fCal). High fECP levels nevertheless did correlate with a necessity for treatment alteration or surgical intervention indicating fECP could be used as a diagnostic tool for remission of the IBD patients (120). Furthermore, increased ECP and MBP deposition was demonstrated in the small bowel of eosinophilic gastroenteritis patients and correlated with disease severity (118). Even though reports have shown increased ECP levels during active inflammation in IBD and related conditions, a causal relationship and conclusive evidence for ECP as a mediator in inflammation is lacking.

#### 3.2.3 Eosinophil Peroxidase (EPO)

The toxic cationic EPO forms hypohalous, hypobromous and hypochlourous acid by using hydrogen peroxide, halide ions and bromide *via* the formation of these acids, EPO can cause cellular damage (121, 122).

Colonic mucosal biopsies from CD patients and colonic perfusion fluids from UC patients express elevated EPO levels during active disease (123–125). Further evidence suggests EPO to be significantly upregulated in tissue of IBD patients at diagnosis, but decreased again during the disease course (126). EPO causes damage to structures *via* nitrate oxidation, and thereby producing toxic reactive oxygen species (127, 128). These reactive oxygen species have previously been linked to renal inflammation and fibrosis (129). A murine DSS colitis model furthermore revealed EPO release into the lumen of the colon and EPO deficient mice showed amelioration in colitis after induction of experimental colitis *via* DSS, suggesting a potential role of EPO in chronic intestinal inflammation (130).

#### 3.2.4 Eosinophil Derived Neurotoxin (EDN)

Unlike the name would suggest, EDN is not neurotoxic for humans. The protein received its name because intracerebral EDN injection showed neuropathological responses in a murine model (122).

Amcoff et al. reported increased fecal EDN protein levels in UC patients not only during but also three months prior to relapse. Therefore, faecal EDN has been proposed as a biomarker or predictor of relapse (131). This prognostic role for EDN in eosinophil mediated intestinal inflammation has also been suggested in pediatric patients (132). EDN might therefore possibly serve as a diagnostic tool or biomarker for gastrointestinal inflammation. Whether the protein additionally contributes directly to inflammation or fibrosis development is still up for debate.

#### 3.2.5 Eosinophil Major Basic Protein (MBP)

MBP, often called proteoglycan2 (PRG2) is encoded by the *PRG2* gene and has two homologues, MBP1 and MBP2. While MBP1 can be detected in eosinophils, basophils and mast cells, MBP2 is only present in eosinophils (133). Due to its cationic nature, MBP can disturb permeability and cell membrane functioning as well (117).

It is believed MBP directly increases the epithelial layer permeability *via* its toxicity (134). *In vitro* co-culture of eosinophils and epithelial cells decreased the integrity of the epithelial barrier, which has been attributed to MBP (135). *MBP* knock out mice do not develop colitis upon oxazolone exposure, indicating a potential role for MBP in intestinal inflammation (135).

In summary (compiled in **Table 1**), eosinophils have been implicated in the inflammatory process in IBD patients (11). Studies have demonstrated an increased number of activated eosinophils in both active and inactive UC compared to healthy controls (25). As the presence of activated eosinophils was more pronounced in quiescent UC compared to active UC, eosinophils have also been suggested to be involved in tissue repair and remodeling mechanisms (25). Moreover, an increased number of degranulated eosinophils and eosinophil granule protein levels has been demonstrated in tissue samples from IBD patients (136). Peripheral blood eosinophilia was furthermore associated with worse clinical outcomes and more severe disease in UC patients (137, 138). *In vivo*, IL-4 production by eosinophils has been shown to promote colitis in both the chemically induced dextran sodium sulphate (DSS), as well as in the T cell transfer model (139). Even though several reports suggest a role for eosinophils in inflammation, conclusive evidence is lacking and therefore requires further investigation.

**Table 1:** Role of eosinophil activating mediators and compounds from eosinophil specific granules in intestinal inflammation.

	Pre-clinical evidence	Clinical evidence
**IL-4**	◊ IL-4 blocking in IL-10 deficient mice: protected from colitis development (63) ◊ No IL-4Rα: no disease development (73)	◊ UC patients: ↑ *IL-4* expression levels in inflamed mucosa (62) ◊ CD patients: ↓ *IL-4* levels in intestinal tissue due to lower numbers of IL-4 producing cells in mucosal biopsies (74)
	◊ IL-4/IL-13 dual antagonist in oxazolone colitis model (67, 68) - Reduced overall disease activity ◊ IL-4/IL-13 blocking trough a shared receptor (69, 71) - Reduced overall disease severity	
**IL-13**		◊ CD and UC patients: ↑ *IL-13Rα2* in mucosal biopsies (94, 95) ◊ Potential biomarker for anti-TNF non-responsiveness (96) ◊ Clinical trial with Tralokinumab and Anrukinzumab: no therapeutic effects (62)
	◊ {IL-13Rα2 KO model IL-13Rα2 antibody mediated depletion 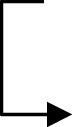 DSS model: mice protected ssssss from colitis introduction (92) ◊ IL-13Rα2 KO model: not protected from colitis development but recovered faster (91)	
**IL-5**		◊ {Mepolizumab & Reslizumab Benralizumab 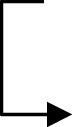 Attenuates type 2 response + used and shown effective in eosnophl eosinophilic asthma patients ◊ UC patients’ rectal perfusion fluids (84): - ↑ IL-5 levels
**IL-33**	◊ SAMP/YitFc colitis model and antibody mediated ST2 blocking (102): - ↓ Th2 cytokine production and ↓ eosinophil recruitment into the ileum ◊ C57BL/6 ST2 KO mice (104) and ST2 antibody mediated depletion in C57BL/6 mice alleviated disease symptoms	◊ UC patients: ↑ colonic *IL-33* mRNA levels and activated eosinophils (100) ◊ IBD patients’ intestinal biopsies (101–103): - ST2/IL-33 signaling - Eosinophil infiltration which coincided with Th2 mediated immune response - IL-4, IL-5 and IL-13 release
**TGF-β1**	◊ TGF-β1 deficient mice: spontaneously develop colitis (113, 114)	◊ Active inflammation in IBD patients: ↑ }TGF-β1 protein levels ↓ (111, 112) =
**EDN**		◊ UC patients: ↑ f(EDN) protein levels during and 3 months prior to relapse: possible prognostic role (131) ◊ Suggested as a prognostic marker in paediatric patients (132)
**ECP**		◊ Active CD and UC: ↑ serum ECP compared to HC (118)
		◊ Eosinophil gastroenteritis: ECP and MBP deposition in small bowel (119)
**MBP**	◊ MBP KO mice: no colitis development upon oxazolone exposure (135) ◊ *In vitro* co-culture of eosinophils and epithelial cells decreased functioning of the epithelial barrier - attributed to MBP (135)	
		◊ MBP directly increases epithelial layer permeability *via* its toxicity (134)
**EPX**	◊ DSS colitis model: ↑ EPX release in colonic lumen (130) ◊ EPX^-/-^ mice: colitis amelioration (130)	◊ CD patients’ colonic mucosal biopsies and UC patients’ colonic perfusion fluids - ↑ EPX levels during active disease (123–125) ◊ IBD patients: EPX ↑ at diagnosis but decreased again during disease course (126)

## 4 Role of Eosinophils in Fibrosis

Because eosinophil infiltration was already shown in other fibrotic diseases, such as endomyocardial fibrosis, idiopathic retroperitoneal fibrosis and pulmonary fibrosis, targeting eosinophils may prove to be beneficial in a number of other fibrotic implications (89, 140). Little is known, however, about the eosinophilic involvement in the development of intestinal fibrosis in IBD. Therefore, the exact mechanism or functioning of the eosinophils in these fibrotic diseases requires further investigation. The involvement of the previously described eosinophil activators and proteins from eosinophil granules in fibrosis will be described below.

### 4.1 IL-4

IL-4, a potent inducer of TGF-β1, stimulates fibroblast expression and release of inflammatory cytokines, thereby stimulating inflammation and lung remodeling and repair in chronic asthma patients (141). Increased IL-4 expression has been linked to pulmonary fibrosis. In this study, IL-4 deficient mice developed significantly less pulmonary fibrosis than wt mice. On the other hand, the same study showed that IL-4 did not directly stimulate collagen type I expression and alpha smooth muscle actin (α-SMA) proliferation (142). While IL-4 has been associated with idiopathic pulmonary fibrosis (IPF), hepatic fibrosis and cardiac fibrosis (143–146), little is known about the role of IL-4 in the development of intestinal fibrosis.

### 4.2 IL-5

This cytokine has also been investigated in a variety of chronic fibrotic diseases, such as hepatic fibrosis (89). By using *IL-5* knock out C57BL/6 mice, Reiman *et al.* were able to show a significant reduction in the development of hepatic fibrosis, determined *via* histopathological analysis, suggesting IL-5 is a potent player in this condition (89). The study demonstrated that IL-5 stimulated the Th2 lymphocyte response and indirectly upregulated IL-13, shown to be a key mediator in the development of fibrosis, indicating IL-5 could have both a direct and an indirect effect on eosinophil mediated liver fibrosis (89). The importance of this Th2 response was later demonstrated in experimental models of pulmonary, renal and intestinal fibrosis (147–149). Moreover, anti-IL-5 mediated treatment depleted the intestinal eosinophils, and suppressed the development of radiation induced intestinal fibrosis (RIF) in mice, demonstrating the importance of eosinophils and IL-5 in the development of RIF (150). Research on IL-5 involvement in the development of intestinal fibrosis however is still limited, highlighting the need for additional research to shed light on the exact pathogenesis.

### 4.3 IL-13

IL-13 has also been implicated in several fibrotic diseases such as pulmonary, renal, hepatic and intestinal fibrosis and was identified as a possible inducer of airway remodeling in asthma patients (151–154). IL-13, together with IL-4, is responsible for eosinophil activation and additionally can activate and proliferate fibroblasts (155). This cytokine was shown to promote lung fibrosis, and IPF patients exhibited increased IL-4 and IL-13 receptors on lung fibroblasts (155). IL-13 has also been implicated in intestinal fibrosis. Fibrosis in chronic TNBS treated mice seemed driven by IL-13 *via* TGF-β1 production, and IL-13 blocking resulted in the prevention of intestinal fibrosis (156, 157). Increased IL-4Rα, IL-13Rα1 and IL-13Rα2 levels were found in ileal strictures in CD patients, indicating IL-13 might be involved (62). Even though IL-13 has been implicated in wound repair, tissue remodeling and fibrosis formation, it is not completely understood how it contributes to the development of strictures in CD patients (92). Even if anti-IL13 treatment was not successful to suppress inflammation in UC patients (cf. 2.1.3), its effect on inflammation and especially fibrosis in CD has not been investigated (62).

### 4.4 IL-33

The co-culture of fibroblasts with eosinophils, activated *via* IL-33, led to the production and release of components that were associated with chronic intestinal fibrosis, including TGF-β (25). Activating eosinophils *via* IL-33 and subsequently co-culturing the activated eosinophils with intestinal fibroblasts resulted in the increase of IL-13Rα2, the pro-inflammatory cytokines TNF-α, IL-1β and IL-6 and the chemokines CCL24 and CCL26 (158). The release of these latter two eosinophil chemoattractant molecules possibly results in additional eosinophil recruitment. Co-cultured fibroblasts were isolated and subsequently cultured with IL-13, leading to the production of fibronectin, collagen 1α2 and periostin, which are pro-fibrotic elements. The role of eosinophils in inflammation and fibrosis might therefore be a two-step mechanism (158). Interestingly, IL-33 is also increased in the ileal specimens of paediatric CD patients compared to healthy controls (158).

### 4.5 TGF-β

TGF-β has been shown to stimulate fibrosis in several organs (1, 159–161). This pro-fibrotic cytokine can affect structural airway cells such as fibroblasts, smooth muscle cells and epithelial cells, and has been implicated in fibrotic diseases such as airway remodeling in asthmatic patients (154). It stimulates fibroblast to myofibroblast activation, and thereby fibrosis (154). *In vitro* culturing of mucosal fibroblasts derived from UC patients during active disease showed increased production of both TGF-β1 as TGF-β3, while mucosal fibroblasts derived from CD patients during active disease showed increased production of TGF-β1, but less TGF-β3 (162). Increased TGF-β1 levels are similarly observed in mucosal biopsies from CD patients (163). TGF-β,however, is produced by a subset of cells, such as epithelial cells, fibroblasts and immune cells, therefore not specifically indicating a role for eosinophils (108). Future research in which eosinophils and their secreted products, such as TGF-β, are blocked could further help to unravel the specific role of eosinophil derived TGF-β in fibrosis development.

### 4.6 ECP

This protein has recently been proposed as a possible mediator in tissue remodeling in allergic asthma patients and in patients with eosinophilic esophagitis (164). In the lungs, tissue remodeling occurs *via* collagen and proteoglycan release from the interstitial fibroblasts. Eosinophils, and ECP in particular, further mediate this process by the production and release of TGF-β (164, 165). Additionally, ECP also causes collagen gel contraction and accumulation of intracellular proteoglycan. ECP might therefore have an indirect effect on fibroblast activation (164). However, conclusive evidence is lacking and additional research is necessary. Moreover, a role for ECP in the development of intestinal fibrosis has not yet been described.

### 4.7 EPO

*EPO* knockout mice showed decreased renal fibrosis development (129). These *EPO* knockout mice also show decreased α-SMA expression and collagen I deposition, indicating a possible involvement in fibrosis development (129). Eosinophils, the source of EPO, also accumulated in the renal interstitium of mice with unilateral ureteral obstruction. Pulmonary epithelial cell exposure to both EPO and MBP resulted in increased mRNA levels of *TGF-α, TGF-β1, epidermal growth factor receptor, platelet derived growth factor* and *tenascin* (166). These factors are all associated with fibroblast activation, indicating EPO might be involved in fibrosis development. Again, conclusive evidence is lacking.

## 5 Other Factors Shaping Eosinophil Function

Neutrophil extracellular traps (NETs), a complex mesh of extracellular fibers primarily consisting of neutrophil DNA have been implicated in inflammatory and fibrotic disorders. Thereby, an excess NET production was suggested to be involved in several pulmonary disorders (167). In that context, NETs have been implicated in the activation of lung fibroblasts and differentiation towards myofibroblasts, correlating to an increased collagen and connective tissue growth factor (CTGF) production (168). Similar to what has been proposed in neutrophils, the potential involvement of eosinophil extracellular traps (EET), already indicated to be involved in tissue damage in the airways of patients suffering from asthma, should be further explored in the context of fibrosis development (169).

Additionally, several studies have indicated a link between the microbiome and intestinal eosinophils. Previously, a significantly higher abundance of eosinophils was shown in germ-free mice compared to pathogen-free mice, suggesting that the microbiome dampens eosinophil proliferation (170). Furthermore, when germ-free mice were exposed to a complex microbiome, a significant decrease in eosinophil numbers was shown (170). Moreover, recent data has shown that high eosinophil presence, resulting from helminth infections, can lead to tissue fibrosis (171). The microbiome therefore clearly has a direct effect on eosinophil numbers and possibly eosinophil functioning.

Lastly, as eosinophils are generally present in the GI tract under homeostatic conditions, they are believed to have a beneficial role in the maintenance of tissue homeostasis. This beneficial role is believed to occur *via* the preserving of IgA producing plasma B cells, thereby promoting Peyer’s patch development and regulating intestinal microbiota composition. Furthermore, eosinophils are considered to enhance intestinal mucus secretion, thereby supporting the epithelial barrier integrity. Lastly, eosinophils have been described to secrete the IL-1 receptor antagonist IL-1Rα thereby inhibiting IL-1β production resulting in decreased Th17 differentiation. As Th17 cells are the main producers of the profibrotic cytokine IL-17A, eosinophils can fulfill an anti-fibrotic role as well (172).

## 6 Treatment Options: Targeting Eosinophils in IBD

At diagnosis, patients with IBD are often treated with corticosteroids in a tapering schedule to quickly improve symptoms (**Table 2**) (189, 190). Corticosteroids prevent eosinophil accumulation, reduce eosinophil chemotaxis and can block other eosinophil factors, including *in vitro* eosinophil adherence (173, 174). Corticosteroids also have a known anti-fibrotic function by reducing collagen synthesis, which is also related to their negative effect on wound healing (175). This anti-fibrotic effect has been demonstrated in several diseases such as idiopathic pulmonary fibrosis, systemic sclerosis and retroperitoneal fibrosis (176–180). However, long-term corticosteroid exposure is not recommended due to systemic side effects (189).

**Table 2:** Conventional treatment options for IBD patients.

Treatment	Influence on eosinophil presence	Influence on fibrostenosis development
**Corticosteroids**	Prevent eosinophil accumulation and reduce eosinophil chemotaxis and can block other eosinophil factors (173, 174).	Demonstrated in idiopathic pulmonary fibrosis, systemic sclerosis and retroperitoneal fibrosis: affects wound healing and reduces collagen synthesis (175–180).
**Anti-α4β7 integrin (Vedolizumab)**	Possible ↓ in influx of eosinophils, but inconclusive evidence (181) ◊ Vedolizumab: no effect on eosinophil circulation ◊ Natalizumab: ↑ in circulating eosinophils ↓ eosinophil accumulation at site of inflammation	No effects described in literature.
**Anti-TNF (infliximab and adalimumab)**	No effect described in literature.	Infliximab: suggested to be effective in the early stages of fibrosis development - ↓ in bFGF and VEGF levels in serum (182). - *In vitro* exposure of myofibroblasts, isolated from CD patients, to infliximab: ↓ collagen production (183). Adalimumab: CREOLE study - CD patients with small bowel strictures: beneficial effect (184)
**Anti-IL-12/IL-23 (Ustekinumab)**	No effect described in literature.	No effect described in literature.
**JAK inhibitor (Tofacitinib)**	Effective in several eosinophil related disorders (185–187) - ↓ in eosinophil numbers - ↓ in disease symptoms	
	BAL (Bronchoalveolar lavage) fluid in mice treated with Tofacitinib (188): - eosinophil presence reduced (188). - ↓ in [TGF-β] - ↓ myofibroblasts deposited in pulmonary arteries	

The IBD therapeutic landscape has changed entirely with the availability of several biological agents and small molecules in the past two decades (**Table 2**). However, the direct effect of these biologicals on eosinophil presence, activation and degranulation is still largely unknown. Mucosal addressin cell adhesion molecule 1 (MadCAM-1), expressed on the vascular endothelium in the intestinal tract, will bind α4β7-integrin, present on the eosinophil surface. This α4β7-integrin/MadCAM-1 binding causes eosinophilic recruitment to the GI tract (191). It would therefore be expected that anti-α4β7-integrin treatment would affect intestinal eosinophil recruitment. However, only inconclusive evidence is available in literature: while Bochner and colleagues reported no effect on eosinophil circulation after vedolizumab treatment (181), natalizumab, a humanized anti-α4β1 and α4β7-integrin antibody approved for treatment of systemic sclerosis, caused an increase in circulating eosinophils and a decreased accumulation of eosinophils at the site of inflammation (181). Non-responders to vedolizumab treatment had higher baseline colonic mucosal mean eosinophil counts. Whether this increased baseline eosinophil count could be used as a predictor for non-response to the humanized antibody vedolizumab should be further investigated (192). While no effects of infliximab treatment on eosinophil presence and activation status have been described, infliximab has been suggested to be effective in the early stages of fibrosis development. Patients treated with infliximab, a chimeric antibody targeting TNF-α, exhibited a decrease in serum levels of basic fibroblast growth factor (bFGF) and vascular endothelial growth factor (VEGF) (182). These factors are known to be involved in the development of intestinal fibrosis; bFGF promotes fibroblast proliferation and VEGF stimulates fibroblast activation and ECM synthesis (193, 194). *In vitro* exposure of myofibroblasts, isolated from CD patients’ active lesions, to infliximab moreover reduced collagen production (183). In the CREOLE study, 97 CD patients with small bowel strictures were treated with the human anti-TNF-α therapy adalimumab. Two thirds (63.9%) of CD patients responded successfully (defined as adalimumab continuation without prohibited treatment, endoscopic dilatation or bowel resection) to adalimumab with a sustained response of 45.7% after 3.8 years, indicating that anti-TNF therapy might have a beneficial effect on intestinal strictures (184).

Tofacitinib, the first JAK-inhibitor approved for moderate-to-severe UC, has shown to be an effective therapeutic in several eosinophil related disorders such as hypereosinophilic syndrome, drug-induced hypersensitivity syndrome and eosinophilic esophagitis (**Table 2**) (185–187). In a pulmonary eosinophilic vasculitis model, the eosinophil abundance in BAL fluid was reduced in 8-week-old C57BL/6 mice treated with tofacitinib. Moreover, decreased TGF-β concentrations were measured in the BAL fluid and less myofibroblasts were deposited in the pulmonary arteries, indicating tofacitinib might not only affect eosinophil infiltration, but could also serve as an anti-fibrotic treatment (188). Nevertheless, tofacitinib failed phase II drug development in patients with luminal CD, and thus no further investigation is scheduled (195). In contrast, the JAK-1 inhibitor filgotinib did show promising efficacy in CD, including a significant decrease in VEGF (196, 197).

Because the eosinophilic role in inflammation and fibrosis is still so little understood, treatments specifically targeting eosinophils are not currently used in IBD patients. Treatments targeting eosinophils in murine models of colitis, however, have shown to decrease inflammation and tissue architecture remodeling (158). Targeting CCR3 or eotaxin seems a potential therapeutic option because of its role in the accumulation of eosinophils, and indeed reduced inflammation in the Samp1/SkuS1c mouse model (198, 199). Targeting eotaxin-1 *via* an anti-eotaxin-1 monoclonal antibody (mAb) in a chemically induced model of colitis also reduced the overall disease severity, and has proven its efficacy in a RAG1 deficient mouse model of allergic inflammation (200–202). Bertilimumab, a human anti-eotaxin-1 mAb was initially developed for the treatment of allergic disorders (203, 204). The same mAb demonstrated a clear beneficial effect in a DSS colitis, suggesting that it should be considered for development in the treatment of IBD (205).

Benralizumab, a humanized anti-IL-5R mAb causing eosinophil depletion, already proved its efficacy in asthma patients (206). A similar mAb was designed and showed to significantly ameliorate radiation-induced intestinal fibrosis in mice, and could therefore be a potential therapeutic target in a specific subset of IBD patients (150).

Targeting the ST2/IL-33 pathway might alleviate disease symptoms for IBD patients. Several IL-33 blocking antibodies are currently under evaluation for asthma and for chronic obstructive pulmonary disease (COPD). Interfering with ST2, however, should be handled with caution because ST2 is involved in the activation of other cell types such as ILC2s, T lymphocytes, mast cells, basophils and several other immune cells and could thereby indirectly affect other pathways (98).

Lastly, as previously mentioned, a study revealed severe eosinophil infiltration in the lamina propria of colonic biopsies to be the most significant predictor of poor response to medical therapy in UC patient, highlighting once more the importance of eosinophil monitoring in IBD patients (15).

## 7 Conclusion

Eosinophils and their granular components have been suggested as pivotal players in several inflammatory and fibrotic diseases including IBD. Although various important mediators of eosinophil recruitment and activation are upregulated in IBD patients, an exact pathogenesis or mechanism through which eosinophils would fulfill their function is still not clear. The specific contribution of eosinophil derived proteins, i.e. ECP, EPO, EDN and MBP, is even less understood. However, pro-fibrotic TGF-β released from eosinophils could potentially contribute to intestinal fibrosis in IBD. Published studies mainly provide descriptive data, rather than demonstrating a clear causative role. Further research will therefore be needed in order to determine the role of eosinophil activation and degranulation in inflammation and fibrosis, specifically in the intestine, and to possibly identify novel anti-inflammatory and anti-fibrotic treatments in IBD.

## Author Contributions

The original draft was written by IJ. All authors contributed equally in the conceptualization and revising. All authors approved the final version of the review.

## Funding

IJ is supported by a KU Leuven grant (ZKD2906-C14/17/097). CB and BV are supported by the Clinical Research Fund KOOR (University Hospitals Leuven, Leuven, Belgium). TV is a Senior Clinical Investigator and LW a doctoral researcher supported by the Research Foundation Flanders (FWO), Belgium.

## Conflict of Interest

CB reports consultancy fees from Ablynx. SV reports financial support for research from MSD, AbbVie, Takeda, Pfizer, J&J, lecture fees from MSD AbbVie, Takeda, Ferring, Centocor, Hospira, Pfizer, J&J, Genentech/Roche, consultancy fees from MSD, AbbVie, Takeda, Ferring, Centocor, Hospira, Pfizer, J&J, Genentech/Roche, Celgene, Mundipharma, Celltrion, Second Genome, Prometheus, Shire, Prodigest, Gilead and Galapagos. BV reports financial support for research from Pfizer lecture fees from Abbvie, Biogen, Chiesi, Falk, Ferring, Galapagos, Janssen, MondayNightIBD, MSD, Pfizer, R-Biopharm, Takeda and Truvion, consultancy fees from Applied Strategic, Atheneum, Bristol Myers Squibb, Guidepont, Ipsos, Janssen, Progenity, Sandoz and Takeda. TV reports financial support for research from Danone and MyHealth, has served on the Speaker bureau for Abbott, Fresenius Kabi, Kyowa Kirin, Menarini, Remedus, Takeda and Will Pharma, consultancy fees from Baxter, Dr. Falk Pharma, Takeda, Tramedico, Truvion, VectivBio and Zealand Pharma.

The remaining authors declare that the research was conducted in the absence of any commercial or financial relationships that could be construed as a potential conflict of interest.

## Publisher’s Note

All claims expressed in this article are solely those of the authors and do not necessarily represent those of their affiliated organizations, or those of the publisher, the editors and the reviewers. Any product that may be evaluated in this article, or claim that may be made by its manufacturer, is not guaranteed or endorsed by the publisher.

## Glossary

**Table:**

α-SMA	alpha smooth muscle actin
ATGL1	Autophagy related protein like 1
BAL	Bronchoalveolar lavage
bFGF	basic fibroblast growth factor
CCL	C-C motif ligand
CCR	C-C chemokine receptor
CD	Crohn’s disease
CD	Cluster of differentiation
CTGF	Connective tissue growth factor
CXCR	C-X-C chemokine receptor
DSS	Dextran sodium sulphate
ECM	Extracellular matrix
ECP	Eosinophil cationic protein
EDN	Eosinophil derived neurotoxin
EET	Eosinophil extracellular traps
EPO	Eosinophil peroxidase
fECP	faecal eosinophil peroxidase
fCal	faecal calprotectin
GI	Gastrointestinal
GM-CSF	Granulocyte-macrophage colony-stimulating factor
IBD	Inflammatory bowel disease
IFN	Interferon
IL	Interleukin
ILC	Innate lymphoid cell
IPF	Idiopathic pulmonary fibrosis
IRGM	Immunity related GTPase M
JAK	Janus kinase
mAb	Monoclonal antibody
MadCam	Mucosal addressin cell adhesion molecule
MBP	Eosinophil major basic protein
MCP	Monocyte chemoattractant protein
mRNA	messenger ribonucleic acid
NETs	Neutrophil extracellular traps
NK	Natural killer
NKT	Natural killer T
NOD-2	Nucleotide-binding oligomerization domain-containing protein 2
PRG2	Proteoglycan 2
RANTES	Regulated upon activation, normal T cell expressed and secreted
RIF	Radiation induced fibrosis
ST2	Suppression of tumorigenicity 2
STAT	Signal transducer and activator of transcription
TGF	Transforming growth factor
TNBS	Trinitrobenzene sulfonic acid
TNF	Tumor necrosis factor
Th	T helper
UC	Ulcerative colitis
VEGF	Vascular endothelial growth factor
wt	wild type
